# Network of vascular diseases, death and biochemical characteristics in a set of 4,197 patients with type 1 diabetes (The FinnDiane Study)

**DOI:** 10.1186/1475-2840-8-54

**Published:** 2009-10-06

**Authors:** Ville-Petteri Mäkinen, Carol Forsblom, Lena M Thorn, Johan Wadén, Kimmo Kaski, Mika Ala-Korpela, Per-Henrik Groop

**Affiliations:** 1 Folkhälsan Institute of Genetics, Folkhälsan Research Center, Biomedicum Helsinki, Finland; 2 Division of Nephrology, Department of Medicine, Helsinki University Central Hospital, Finland; 3 Department of Biomedical Engineering and Computational Science, Helsinki University of Technology, Finland; 4 Computational Medicine Research Group, Institute of Clinical Medicine, Faculty of Medicine, University of Oulu and Biocenter Oulu, Finland; 5 Department of Internal Medicine and Biocenter Oulu, Clinical Research Center, University of Oulu, Finland; 6 The Baker IDI Heart and Diabetes Institute, Melbourne, Victoria, Australia

## Abstract

**Background:**

Cardiovascular disease is the main cause of premature death in patients with type 1 diabetes. Patients with diabetic kidney disease have an increased risk of heart attack or stroke. Accurate knowledge of the complex inter-dependencies between the risk factors is critical for pinpointing the best targets for research and treatment. Therefore, the aim of this study was to describe the association patterns between clinical and biochemical features of diabetic complications.

**Methods:**

Medical records and serum and urine samples of 4,197 patients with type 1 diabetes were collected from health care centers in Finland. At baseline, the mean diabetes duration was 22 years, 52% were male, 23% had kidney disease (urine albumin excretion over 300 mg/24 h or end-stage renal disease) and 8% had a history of macrovascular events. All-cause mortality was evaluated after an average of 6.5 years of follow-up (25,714 patient years). The dataset comprised 28 clinical and 25 biochemical variables that were regarded as the nodes of a network to assess their mutual relationships.

**Results:**

The networks contained cliques that were densely inter-connected (*r *> 0.6), including cliques for high-density lipoprotein (HDL) markers, for triglycerides and cholesterol, for urinary excretion and for indices of body mass. The links between the cliques showed biologically relevant interactions: an inverse relationship between HDL cholesterol and the triglyceride clique (*r *< -0.3, *P *< 10^-16^), a connection between triglycerides and body mass via C-reactive protein (*r *> 0.3, *P *< 10^-16^) and intermediate-density cholesterol as the connector between lipoprotein metabolism and albuminuria (*r *> 0.3, *P *< 10^-16^). Aging and macrovascular disease were linked to death via working ability and retinopathy. Diabetic kidney disease, serum creatinine and potassium, retinopathy and blood pressure were inter-connected. Blood pressure correlations indicated accelerated vascular aging in individuals with kidney disease (*P *< 0.001).

**Conclusion:**

The complex pattern of links between diverse characteristics and the lack of a single dominant factor suggests a need for multifactorial and multidisciplinary paradigms for the research, treatment and prevention of diabetic complications.

## Background

A significant number of patients with type 1 diabetes suffer from severe microvascular complications such as diabetic kidney disease and proliferative retinopathy [1,2]. The pathogenetic mechanisms responsible for the degradation of the vascular system are not yet fully known, but a complex pattern of interactions between susceptibility genes and environmental factors is the likely cause. Kidney failure is not the primary cause of death, but these patients die mostly from cardiovascular complications at the later stages of the disease [1,3,4]. The risk factors have been extensively investigated [5-7]; however, only a handful of studies have focused on the statistical associations between biochemical and clinical variables from a multivariate perspective [8-10]. The biological variation at the individual level is substantial, which means that the phenotype cannot be compressed into a single variable. Albuminuria, for instance, is the most important clinical risk factor, but it alone provides only limited information on the systemic changes in the body.

Complex network analysis has gained popularity as new datasets and techniques have become available [11,12]. Recent examples include communication patterns in social networks [13,14], molecular interactions in proteomics and metabolomics [15,16] and the epidemiology of contagious diseases [17,18]. Visualization of the network structures helps to understand the complex phenomena and computerized applications are commonplace in network research [19,20].

This work illustrates the main modules of clinical and biochemical associations in type 1 diabetes. Our aim is to present the characteristics of diabetic complications as an inter-connected system, instead of focusing on any single variable at a time. We also discuss the biological processes that can be attributed to the observed network structures, and demonstrate the links between multiple chronic conditions, lifestyle, aging, and metabolic traits in their full context.

## Methods

Type 1 diabetic patients were recruited by the Finnish Diabetic Nephropathy Study Group (N = 4,197). The design was cross-sectional (serum and urine samples), but with longitudinal records of albuminuria and clinical events before baseline and with all-cause mortality data available after an average of 6.5 years of follow-up from baseline (25,714 patient-years). Type 1 diabetes mellitus was defined as an age of onset below 35 years and transition to insulin treatment within a year of onset. Macrovascular disease (337 cases) was obtained from medical records and defined as a pooled end-point of coronary heart disease (224 cases), myocardial infarction (124 cases), stroke (100 cases), and peripheral vascular disease (91 cases).

The classification of renal status was made centrally according to urinary albumin excretion rate (AER) in at least two out of three consecutive overnight or 24 h-urine samples. Absence of diabetic kidney disease (DKD) was defined as AER within the normal range (AER <20 μg/min or <30 mg/24 h) and at least 15 years of type 1 diabetes. This kidney disease negative subset is denoted by 'KDNEG'. Macroalbuminuria or overt kidney disease was defined as AER ≥ 200 μg/min or ≥ 300 mg/24 h. The intermediary range was defined as microalbuminuria (20 ≥ AER <200 μg/min or 30 ≥ AER <300 mg/24 h). Patients on renal replacement therapy (dialysis or transplantation) were classified as having end-stage renal disease (ESRD). An additional subset, denoted by 'DMDur<15', was formed from patients with less than 15 years of diabetes duration, and normal (1,004 individuals) or unknown AER (135 individuals). A total of 296 patients could not be classified.

The AER values that were used for the DKD diagnosis were measured in the local health care centers, but not used for statistical analyses. Instead, the continuous 24 h albumin excretion rate was estimated from a single 24 h-urine collection (available for 80% of patients) from which albumin was measured by a central laboratory.

Education level, smoking and alcohol dose, working status, asthma, rheumatoid arthritis and thyroid disease were determined by patient questionnaires. Education level was defined as the expected number of years in the educational system based on the current occupation, smoking exposure was calculated as the product of daily cigarettes and years of smoking, the daily dose of alcohol was estimated from the type and quantity of drinks consumed. Working status was compressed into a binary trait (disabled vs. employed or unemployed). Serum concentration of the soluble receptor for advanced glycation end-products (SRAGE) was measured by solid phase ELISA (Thomas *et al*. submitted). VLDL triglycerides and IDL and LDL cholesterol were estimated by neural network modeling [21]. Other details on the data sources, clinical definitions and patient characteristics have been published previously [22]. More information on the kidney disease subsets is available in [Additional file 1].

### Statistical analysis

Many of the continuous variables had skewed distributions and it is typical for a large clinical study to have a small percentage (<5%) of outliers. Therefore, the continuous variables were sorted and converted to scaled ranks between -1 and 1 to prevent statistical artifacts. Two versions of the dataset were created: one with men and women pooled, the other with separate rank transforms for the sexes.

The network of continuous variables was based on pair-wise Spearman's correlation coefficients. Specifically, each variable is considered a node and the nodes are connected by links, the weights of which are quantified by the correlation coefficient. The full networks are too dense and have to be pruned in order to highlight the relevant patterns. There are numerous ways to reduce the network dimensionality [23], here we chose the spanning trees since they are computationally efficient and ensure the connectivity of the pruned network [24].

Direct graph-theoretic investigation of the networks provides little useful information since many of the variables are derived or otherwise non-biologically linked with each other. Therefore, structural considerations were made via comparisons between the kidney disease subsets to reduce the distraction from irrelevant connections. Statistical significance was estimated by random permutations of the subset labels [Additional file 2]. Although the individual links are not independent, they may be subject to multiple testing effects (less than 741 tests). P-values between 0.01 and 0.0001 are therefore considered suggestive. Topologically relevant links were chosen as follows: i) the link must belong to at least one of the spanning trees from difference networks between KDNEG and the other subsets and ii) the link must be one of the top 10 most significant (and *P *< 0.01) in its spanning tree. This procedure was chosen to avoid selecting too many links for closer inspection, and yet ensuring that as many nodes as possible would be represented.

Correlation coefficient is not well suited for comparing binary and continuous variables. For this reason, a computationally intensive regression-correlation measure was applied to the full dataset to create the visualization [Additional file 2]. All statistical analyses were performed with in-house scripts in the Octave programming environment .

## Results

**Figure 1:**
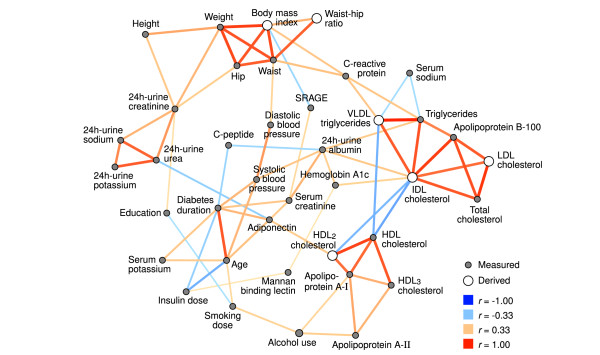
**Correlation network of continuous data**. A pruned visualization of the correlation structure within a set of patients with type 1 diabetes. Prior to the analysis, the data were adjusted for gender. Each variable is presented with a symbol; those quantities that were measured directly are filled with ink and the open circles denote derived variables. The width and color of the links indicate the correlation magnitude and type, as shown in the legend. The *r *denotes Spearman correlation and SRAGE is abbreviation for soluble receptor for advanced glycation end-products. Visualized with the Himmeli software [47].

Figure 1 depicts the correlation structure of the gender-adjusted dataset. The network is characterized by strong links between methodologically and biochemically dependent variables: markers of body mass (weight, BMI, WHR, etc.), 24 h-urine excretion (potassium, urea, sodium and creatinine), HDL-related biochemistry (HDL cholesterol, apolipoprotein A-I and A-II) and other lipoprotein quantities (triglycerides, total cholesterol, apolipoprotein B-100) form positively correlated cliques.

There are strong inverse associations between the HDL-clique and IDL cholesterol. Other connections include the links between triglycerides and body mass via C-reactive protein, and the central role of 24 h-urine albumin as the connector of triglycerides, IDL cholesterol, hemoglobin A1c, blood pressure and serum creatinine. Adiponectin links HDL metabolism with 24 h-urine metabolites and SRAGE is located between serum creatinine and body mass. Smoking and alcohol intake are correlated; alcohol consumption is also reflected in apolipoprotein A-I and A-II concentrations, and smoking dose is linked with lower education and - by definition - to higher age. Weight-adjusted insulin dose is inversely associated with aging in this dataset.

### Network topology and diabetic kidney disease

The dataset was divided according to AER and diabetes duration (see Methods) and the subset networks were compared to detect relevant topological features. Table 1 shows the statistical significance of difference networks (based on the sets of pair-wise correlation coefficients) between the patient groups. The correlation structure for the KDNEG subset with 15 years or more duration is significantly different from the macroalbuminuria subset (*P *= 2.2 × 10^-16^) and from the patients with short duration (*P *= 4.0 × 10^-32^). On the other hand, the DMDur<15 subset is different from the macroalbuminuria subset (*P *= 1.2 × 10^-29^).

**Table 1:** Comparison of diabetic kidney disease networks

	**Microalbuminuria** **n = 508**	**Macroalbuminuria** **n = 586**	**ESRD** **n = 289**	**DMDUR<15** **n = 1,139**
KDNEG n = 1,379	0.0056	2.2 × 10^-16^	4.6 × 10^-27^	4.0 × 10^-32^

Microalbuminuria		7.1 × 10^-6^	7.3 × 10^-18^	7.8 × 10^-20^

Macroalbuminuria			6.2 × 10^-9^	1.2 × 10^-29^

ESRD				8.3 × 10^-31^

Statistical significance estimates (*P*-values) from permutation analysis of difference networks. The networks were formed from pair-wise Spearman correlation coefficients of 39 continuous clinical and biochemical variables.

Table 2 lists the (selected) significant changes in link weights with respect to the KDNEG subset. Age and blood pressure show a mixed trend: diastolic blood pressure has a negligible age-dependence in the KDNEG subset, but an inverse correlation in the macroalbuminuria subset (*r *= 0.02 vs. -0.20, *P *= 2.5 × 10^-5^), whereas systolic blood pressure shows stronger dependence in the KDNEG subset (*r *= 0.43 vs. 0.28, *P *= 4.0 × 10^-4^). Adiponectin is also age-dependent in the KDNEG subset, but uncorrelated in the macroalbuminuria subset (*r *= 0.32 vs. 0.08, *P *= 8.5 × 10^-7^).

**Table 2:** Correlations within diabetic kidney disease groups

	**KDNEG *r***	**Microalbuminuria *r***	**Macroalbuminuria *r***	**ESRD *r***
Age -- Diastolic blood pressure	0.02	-0.15*	-0.20**	-0.29**

Age -- Systolic blood pressure	0.43	0.35	0.28*	0.06**

Adiponectin -- Age	0.32	0.30	0.08**	-0.01**

Adiponectin -- HDL cholesterol	0.45	0.36	0.21**	0.10**

ApoA-II -- HDL_2 _cholesterol	0.13	0.17	0.34**	0.33*

ApoA-II -- Waist	0.16	-0.02*	0.02*	0.05

Total cholesterol -- Education	-0.03	-0.18*	-0.08	-0.01

Serum creatinine -- Adiponectin	0.05	0.03	0.29**	0.18

Serum creatinine -- Diabetes duration	0.07	0.22*	0.17	-0.06

Serum creatinine -- Insulin dose	-0.01	-0.17*	-0.15*	-0.13

Serum creatinine -- SRAGE	0.03	0.05	0.33**	0.40**

Serum creatinine -- 24 h-uAlb	0.06	0.07	0.15	0.44†

CRP -- Age	-0.10	0.09*	0.05*	-0.01

CRP -- Serum potassium	-0.05	0.12*	-0.01	-0.02

CRP -- Waist-hip ratio	0.18	0.34*	0.23	0.22

IDL cholesterol -- LDL cholesterol	0.72	0.63*	0.53**	0.53**

LDL cholesterol -- Education	-0.01	-0.17*	-0.07	0.01

MBL -- 24 h-urine urea	0.08	-0.10*	-0.05	-0.02†

Serum potassium -- Diabetes duration	0.27	0.26	-0.02**	-0.02**

VLDL triglycerides -- 24 h-uAlb	0.07	0.12	0.22*	0.51†

24 h-uAlb -- ApoB	0.07	0.19	0.27**	0.31†

24 h-uAlb -- Total cholesterol	0.02	0.17*	0.23**	0.16†

24 h-uAlb -- HDL cholesterol	-0.06	-0.04	-0.12	-0.46†

24 h-uAlb -- IDL cholesterol	0.06	0.16	0.30**	0.50†

24 h-uAlb -- Triglycerides	0.08	0.13	0.23*	0.50†

24 h-uAlb -- 24 h-urine creatinine	0.11	-0.05*	0.02	-0.36†

24 h-uAlb -- 24 h-urine urea	0.04	-0.06	-0.06	-0.41†

Comparison of the KDNEG subset network against the micro-, macroalbuminuria and ESRD networks. The links were chosen by an automatic network topology algorithm (see Methods for details). The Spearman correlation coefficient (denoted by *r*) of continuous clinical and biochemical variables was used as the measure of association between the variables. Urine samples were not available from most patients with ESRD (72% missing); the *r *values presented were obtained from the imputed dataset. The links are sorted alphabetically. *P < 0.01, **P < 0.0001, comparison with KDNEG; † imputed.

Serum creatinine is connected to adiponectin (*r *= 0.05 vs. 0.29, *P *= 6.3 × 10^-8^) and SRAGE (*r *= 0.03 vs. 0.33, *P *= 1.5 × 10^-10^) in the macroalbuminuria, but not in the KDNEG subset. The associations between albumin excretion and other variables are also negligible in the KDNEG subset. On the other hand, 24 h-urine albumin is significantly correlated with total cholesterol (*r *= 0.02 vs. 0.23, *P *= 3.0 × 10^-6^), IDL cholesterol (*r *= 0.06 vs. 0.30, *P *= 3.0 × 10^-8^) and triglycerides (*r *= 0.08 vs. 0.23, *P *= 3.0 × 10^-4^) in the macroalbuminuria subset.

Node strength measures the overall connectivity of a node: it is the sum of the correlation magnitudes that link the node to the rest of the network. A high strength indicates a structurally significant variable, although the value itself is less important and therefore not reported here. The strengths were not different between the microalbuminuria and KDNEG subsets (data not shown). Within the macroalbuminuria subset, the connections surrounding serum creatinine (*P *= 5.1 × 10^-5^), 24 h-urine albumin excretion (*P *= 0.0010), SRAGE (*P *= 0.0024) and apolipoprotein B-100 (*P *= 0.0046) were significantly changed. The ESRD group showed a similar structure: serum creatinine, 24 h-urine albumin and SRAGE were significantly different (*P *< 0.00050). There were statistically significant differences also in the connectivity of adiponectin (*P *= 8.1 × 10^-6^), age (*P *= 8.3 × 10^-5^), 24 h-urine urea (*P *= 0.00032), BMI (*P *= 0.0021), insulin dose (*P *= 0.0034) and apolipoprotein A-I (*P *= 0.0099) when compared with the KDNEG subset.

### Regression-correlation network

**Figure 2:**
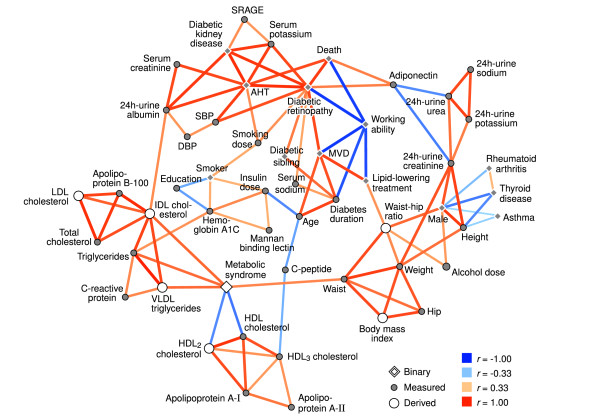
**Regression-correlation network of continuous and binary data**. A pruned visualization of the correlation network from regression modeling. Unlike in Figure 1, the data were not adjusted for gender prior to the analysis. Each variable was converted to a surrogate linear predictor before computations. The symbols in the figure correspond to the source of information: directly observed variables are filled, whereas derived variables are denoted by open symbols. A circle is used for continuous quantities, and a diamond for binary traits. The width and color of the links indicate the association magnitude and type, as shown in the legend. The *r *denotes the correlation of the linear predictors and is not comparable with Figure 1. Abbreviations: history of macrovascular disease (MVD), systolic (SBP) and diastolic (DBP) blood pressure, anti-hypertensive treatment (AHT) and soluble receptor for advanced glycation end-products (SRAGE). Visualized with the Himmeli software [47].

Figure 2 depicts the network based on regression modeling of both the continuous and binary variables. The data were not adjusted for gender effects, since gender was included as a clinical trait. The network is characterized by a high level of connectivity between DKD (with high 24 h-urine albumin and serum creatinine), high blood pressure (and anti-hypertensive treatment), diabetic retinopathy and death. There is a strong link via diabetic retinopathy to old age, long diabetes duration and macrovascular disease, and the same pattern is also reflected by reduced working ability.

Urine metabolites (urea and creatinine) are connected to the complications via adiponectin, and the clique is located next to the body mass indicators (height and weight). Male gender is connected to body mass, as expected, but there are also weak inverse associations with asthma, thyroid disease and rheumatoid arthritis.

The metabolic syndrome is - by definition - a connector between body mass (waist circumference), HDL-metabolism, and triglycerides. The HDL clique is linked to C-peptide an further to age and diabetes duration. On the other hand, age connects to triglycerides via insulin dose and hemoglobin A1c. Finally, the estimated IDL cholesterol is the connector between the triglyceride and cholesterol cliques, and albuminuria.

## Discussion

The network analysis showed identifiable cliques of inter-connected variables that were mostly driven by methodological factors and basic biology. That said, there were biologically relevant links between the cliques: body mass and triglycerides were connected by C-reactive protein in the gender-adjusted analysis, IDL cholesterol was the key quantity between albuminuria, triglycerides, cholesterol, HDL-metabolism and hemoglobin A1c, and the close relationship with working ability and microvascular diseases indicated the debilitating effects of diabetic complications. The lack of correlation between age and systolic blood pressure in the kidney disease patients - but inverse correlation with diastolic - reflected the effects of kidney disease on vascular aging.

Macrovascular disease was not, as could have been expected, the closest to the diabetic kidney disease and blood pressure clique, but the node was located near age and diabetes duration. Nevertheless, the connections to microvascular complications and mortality were evident via retinopathy and reduced working ability. The available data and definitions may also have favored the stronger links with aging: the vascular events were determined from (past) medical records, not at the time of the study visit. There may also be a survival effect: those patients that reach the late stages of kidney disease may have more resilience against cardiovascular disease by having higher HDL cholesterol, for instance [22,25].

Mortality and kidney disease were not connected directly, although part of the same node group. Instead, laser-treated retinopathy and anti-hypertensive treatment had a direct link with death, and there was also a strong inverse connection to working ability (Figure 2). Macroalbuminuria is a powerful risk marker, but these results may indicate the later stages when the patient's health deteriorates to the point were normal life is severely interrupted (loss of sight and working ability) and death ensues. There were twice as many patients without ESRD in the kidney disease group (586 vs. 289). Furthermore, patients with ESRD suffer from secondary effects of kidney failure that disturb the metabolic patterns. This means that the DKD node in the regression-correlation network may be a more accurate estimate for persistent albuminuria (before kidney failure) than for ESRD and death.

The DCCT Study has established the beneficial effects of tight glycemic control on diabetic complications [26,27]. Here, hemoglobin A1c was not among the most structurally significant nodes, although it was suggestively positioned between insulin dose and triglycerides in Figure 1. This does not mean that better insulin treatment is useless; it most likely reflects the biological variability of the A1c measure in our observational data [28]. Advanced glycation end-products in general have been implicated in diabetic tissue damage [29]. The soluble receptor (SRAGE) was connected to complications (kidney function) in this study, although the result can also be explained by reduced clearance.

The patient material was extensive with detailed clinical characteristics and biochemical measurements from serum and urine. On the other hand, the dataset was not complete and special procedures had to be taken to impute the missing values. Many of the correlation coefficients were small (*r *< 0.3) and cannot be considered clinically significant. This is most likely due to the robust but less sensitive rank transform, the need to avoid linear artifacts in data imputation, and the observational nature of the study. In particular, 24 h urinary albumin excretion was weakly correlated with the other variables, which may be the result of the large biological variation in cross-sectional urine collections. There were only a few cases of asthma, rheumatoid arthritis or thyroid disease, which further reduced the power of the regression-correlation approach. Despite the problems, the negative associations with male gender were consistent with previous results [30].

Visual inspection of the networks was validated by additional analysis with alternate preprocessing and statistical comparisons of patient subsets. Nevertheless, the figures produced by the automatic graph drawing software are always simplifications of the true situation and should not be used as a basis of inference without their original context. The selection and availability of variables is the critical determinant of the observed network structures, and should be taken into consideration when interpreting the results. Also, random fluctuations can change individual links, but the overall structure of a correlation network is usually resilient against sampling noise. Statistical significance estimates were not available for the regression-correlation network due to its method of construction. Nevertheless, the observations from Figure 2 were consistent with the correlation network in Figure 1, which suggests that the illustrations are reliable.

Skewed or highly variable biomarkers such as 24-h urine albumin and serum creatinine produce a correlation bias due to non-uniform signal-to-noise ratio in the subset comparisons. Low values have proportionally higher measurement errors than higher values, so the results in Table 2 also reflect changes in absolute concentrations. This is not necessarily an undesired effect; the network still reflects biologically relevant phenomena, albeit not pure associations.

The subset analyses were not matched for age. However, the association between age and diabetes duration was comparable between the groups (except within DMDur<15) and the age variances were similar [Additional file 1], which suggests that the differences in correlation coefficients were not produced by limited age ranges. The macroalbuminuria subset had longer duration than the KDNEG (29 vs. 26 years, *P *= 7.7 × 10^-11^) but, although statistically significant, the modest time gap did not interfere with the descriptive nature of the network approach.

Lipid abnormalities have been previously linked with diabetic kidney disease [31-33] and low HDL and high IDL have been implicated in cardiovascular risk [34-36]. In this study, the estimated IDL cholesterol had the strongest link with albuminuria, which is concordant with a mortality analysis of the same dataset [21]. The result may be partly explained by the reduction in the relative measurement noise after combining the three basic lipids (triglycerides, total and HDL cholesterol), but IDL was nevertheless the most important among the derived lipoprotein variables.

Low-grade chronic inflammation has emerged as a possible link between obesity and insulin resistance. For instance, when adipose tissue expands to accommodate excess lipids, macrophages therein are exposed to non-esterified fatty acids and respond by increased release of inflammatory cytokines [37,38]. The cytokines, in turn, disrupt the normal insulin signaling and fatty acid metabolism in the skeletal muscle. In the gender-adjusted network, C-reactive protein (a marker of inflammation) was positioned between the body-mass and the triglyceride cliques (Figure 1), thus reflecting the underlying biological mechanisms. In the regression-correlation network, C-reactive protein was connected with triglycerides only, probably due to the stronger link between gender and body mass (Figure 2).

Serum adiponectin is another signaling molecule that can be traced to adipose tissue - it is decreased in obesity and insulin resistance [39]. On the other hand, clinical research indicates that in kidney disease the concentration is increased [40,41] despite the simultaneous reduction in insulin sensitivity [22,42]. In this study, adiponectin was positioned as the connector between death and microvascular complications, and 24 h-urine metabolites (Figure 2), and a similar role in the middle of aging, serum creatinine and urine excretion remained in the gender-adjusted network (Figure 1). The results suggest that kidney function is a stronger determinant of adiponectin concentrations in these patients than the inverse correlation with obesity.

Aging and diastolic blood pressure are first positively correlated but then become negatively correlated at higher age. The process is accelerated in type 1 diabetes, most likely due to arterial stiffening [43,44]. The same phenomenon was also detected here from another perspective: significantly stronger negative correlations were observed within the macroalbuminuria and ESRD groups, which can indicate that the vascular aging is ahead of the KDNEG subset, even beyond the small chronological age difference. On the other hand, systolic blood pressure is less age-dependent in macroalbuminuria and ESRD groups, which is probably caused by interference from medication and the decline in the capacity of the heart to compensate for arterial stiffening [45,46].

## Conclusion

The various clinical and biochemical risk factors that predispose to cardiovascular disease and diabetic complications share mutual connections that have overlapping origins in methodology, physiology and pathology. It may not be possible to fully isolate the effects of the various components in the traditional reductionist framework. Therefore, we think that the complex pattern of links between diverse characteristics such as working ability, life style, aging and biofluid chemistry is explicit evidence to develop multifactorial and multidisciplinary paradigms for the research, treatment and prevention of diabetic complications.

## Competing interests

The authors declare that they have no competing interests.

## Authors' contributions

VPM designed and conceived the study, wrote the statistical software, analyzed the data and wrote the manuscript. CF collected the clinical data and reviewed the manuscript. LT collected the clinical data and reviewed the manuscript. JW collected the clinical data and reviewed the manuscript. KK participated in conceiving the study and reviewed the manuscript. MAK participated in conceiving the study and in writing the manuscript, PHG participated in conceiving the study, in collecting the clinical data, and in writing the manuscript.

## Supplementary Material

Additional file 1 **Comparison of kidney disease groups**. Median values (50% quantile) and 68% intervals (equivalent to ± SD for normally distributed variables) of the five patient subsets. The Kolmogorov-Smirnov test was used for continuous data. The P-values were obtained by comparing a given subset against the KDNEG group. Click here for file

Additional file 2 **Network methodology**. A description of the statistical and visualization methods that were used in the study. Click here for file

Additional file 3 **The Finnish Diabetic Nephropathy Study Group**. A listing of the hospitals and health care centers that have participated in the recruitment of patients. Click here for file
