# Supplementary material for: Network of vascular diseases, death and biochemical characteristics in a set of 4,197 patients with type 1 diabetes (The FinnDiane Study)
Source: Cardiovasc Diabetol. 2009 Oct 6;8:54. doi: 10.1186/1475-2840-8-54 (PMC2763862; doi:10.1186/1475-2840-8-54)
Supplement: Additional file 1 — Comparison of kidney disease groups. Median values (50% quantile) and 68% intervals (equivalent to ± SD for normally distributed variables) of the five patient subsets. The Kolmogorov-Smirnov test was used for continuous data. The P-values were obtained by comparing a given subset against the KDNEG group. [file 1475-2840-8-54-S1.PDF]

Additional file 1

	Unit	KDNEG		Microalbuminuria		Macroalbuminuria		ESRD		DMDur<15	
		q_{50}	q_{16} - q_{84}	q_{50}	q_{16} - q_{84}	q_{50}	q_{16} - q_{84}	q_{50}	q_{16} - q_{84}	q_{50}	q_{16} - q_{84}
Age	year	42	30 - 53	38**	27 - 51	41	32 - 51	44**	36 - 53	27**	20 - 37
DMDur	year	26	18 - 37	26**	15 - 38	29**	21 - 37	32**	24 - 41	9**	3 - 13
Male		43%		59%**		58%**		61%**		55%**	
MVD		6%		6%		15%**		37%**		<1%**	
MetS		24%		34%**		56%**		64%**		22%	
DRP		25%		48%**		80%**		98%**		1%**	
TX		0%		0%		0%		75%**		0%	
AHT		20%		65%**		94%**		91%**		5.6%	
DBP	mmHg	78	70 - 87	80	70 - 90	82	72 - 92	87**	74 - 97	78	69 - 87
SBP	mmHg	130	116 - 148	135	120 - 152	144**	125 - 165	150**	130 - 175	125**	112 - 139
WHR		0.85	0.77 - 0.93	0.88**	0.80 - 0.97	0.90**	0.81 - 0.99	0.93**	0.84 - 1.01	0.85	0.77 - 0.92
Adiponectin	mg/l	12.2	7.4 - 18.5	11.2	7.3 - 18.5	15.6**	9.0 - 27.7	20.3**	12.3 - 36.6	9.9**	6.6 - 14.9
Apo-B	mg/dl	82	64 - 104	90	70 - 111	100**	79 - 123	98*	74 - 124	81*	63 - 105
Cholesterol	mmol/l	4.8	4.0 - 5.7	5.0	4.1 - 5.9	5.3**	4.4 - 6.4	5.3**	4.3 - 6.5	4.5*	3.8 - 5.5
sCreat	µmol/l	81	65 - 96	87*	71 - 107	130**	88 - 260	148**	95 - 645	80	65 - 95
A1c	%	8.1	7.0 - 9.3	8.6	7.3 - 10.0	8.8*	7.6 - 10.3	8.6	7.1 - 10.1	8.0**	6.7 - 9.7
HDL-C	mmol/l	1.35	1.03 - 1.79	1.28**	0.96 - 1.67	1.16**	0.83 - 1.54	1.14**	0.78 - 1.63	1.28*	1.00 - 1.66
IDL-C	mmol/l	0.20	0.15 - 0.28	0.23	0.16 - 0.33	0.28**	0.19 - 0.41	0.29**	0.20 - 0.45	0.20	0.14 - 0.28
SRAGE	ng/ml	1.09	0.74 – 1.56	1.10	0.70 – 1.57	1.38**	0.87 – 2.25	1.25**	0.78 – 2.78	1.21*	0.82 – 1.69
TG	mmol/l	0.90	0.60 - 1.40	1.06	0.72 - 1.84	1.40**	0.87 - 2.53	1.49*	0.91 - 2.59	1.01*	0.67 - 1.70
24h-uAlb	mg	8	4 - 17	60**	18 - 148	602**	134 - 2441	67**	12 - 1097	8	4 - 17

*$P < 0.01$, **$P < 0.0001$, comparison with KDNEG group

Abbreviations

	Description		Description
DMDur	type 1 diabetes duration	sCreat	serum creatinine
MVD	history of macrovascular disease	A1c	glycated hemoglobin
MetS	metabolic syndrome by modified NCEP ATP III	HDL-C	serum high-density lipoprotein cholesterol
DRP	history of diabetic retinopathy	IDL-C	estimated serum intermediate-density lipoprotein cholesterol
TX	kidney transplant	SRAGE	serum soluble receptor for advanced glycation end-products
AHT	anti-hypertensive treatment	TG	serum total triacylglycerols (triglycerides)
DBP	diastolic blood pressure	24h-uAlb	24h-urine albumin excretion rate from a single collection
SBP	systolic blood pressure	q	quantile
WHR	waist-to-hip ratio		
Apo-B	apolipoprotein B-100		
