# Supplementary material for: Network of vascular diseases, death and biochemical characteristics in a set of 4,197 patients with type 1 diabetes (The FinnDiane Study)
Source: Cardiovasc Diabetol. 2009 Oct 6;8:54. doi: 10.1186/1475-2840-8-54 (PMC2763862; doi:10.1186/1475-2840-8-54)
Supplement: Additional file 2 — Network methodology. A description of the statistical and visualization methods that were used in the study. [file 1475-2840-8-54-S2.PDF]

Additional file 2

A typical medical dataset is a mixture of binary, categorical and continuous variables. From a technical perspective, full analysis of such data requires careful crafting of dedicated statistical models (hierarchical models, Bayes networks, structural equations, decision trees, etc.). Unfortunately, clinical studies often suffer from a range of confounding effects such as differences in medication guidelines over the years, changes in diagnostic criteria and laboratory protocols and uneven sampling of the base population. It is therefore cost-effective to first use unsupervised methods to rapidly describe the basic properties of the dataset before making more specific hypothesis and model construction.

In this study, the emphasis is on the correlations between variables: each variable is considered a node and the nodes are connected by links, the weights of which are quantified by the strength of statistical association (Figure S1). Patient profiles were already investigated by the self-organizing map (Mäkinen VP *et al.* Diabetes 57 (2008) 2480-2487), but that study did not address the dependencies between the various risk factors. Therefore, an unsupervised network approach was chosen to uncover the patterns of associations. Networks are also appealing due to their ability to describe multi-body interactions, that is, the simultaneous depiction of the mutual relationships between multiple traits were of interest.

Statistical significance

It is obvious that methodologically and physiologically linked variables will show clear patterns in the resulting correlation network. For example, the Friedewald LDL cholesterol is calculated from triglycerides, total cholesterol and HDL cholesterol, and people with a larger body mass will excrete more urine metabolites in a given time unit. Consequently, it makes little sense to investigate the statistical significance of the network structures *per se* since the trivial connections will mask any interesting biological patterns.

In this study, structural considerations were made via comparisons between the kidney disease subsets to reduce the distraction from irrelevant connections. Before going further, however, some terminology is needed. The term spanning tree refers to a selection of links that connects every node with the least possible number of links, while maximizing the link weights (Figure S1) and subset network refers to a network constructed from a subset of patients. A difference network is defined as the subtraction of correlation coefficients (i.e. link weights) between two subset networks, one node pair at a time.

Comparisons of subset networks (formed according to the kidney disease status) were validated by permutation analysis, as summarized in Figure S2. First, two subsets were selected from the dataset, designated as cases and controls. Next, the difference network was calculated (observation). Then the null distribution of differences was simulated by randomly shuffling the case-control labels 10,000 times (effectively creating a large number of random subsets), and each time recording the differences. Finally, the observation was compared with the simulated distribution to obtain an estimate for P-value.

Topologically significant links were chosen as follows: i) the link must belong to at least one of the spanning trees from difference networks between KDNEG and the other groups and ii) the link must be one of the top 10 most significant in its spanning tree. This procedure was chosen to avoid selecting too many links for closer inspection, and yet ensuring that as many nodes as possible would be represented.

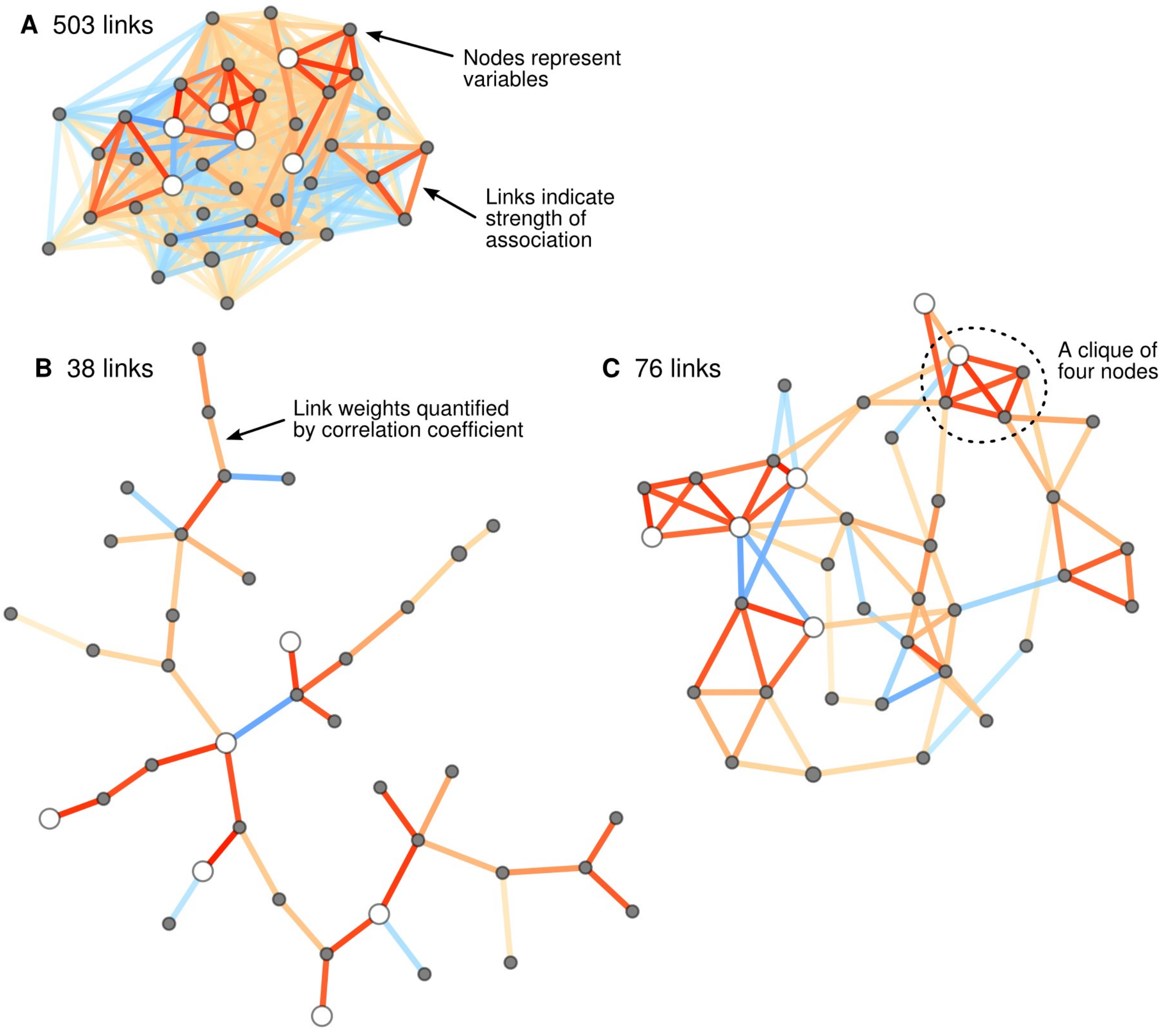

Figure S1: Effect of pruning by spanning links on a small dense network. **A**: Original network with all detectable links visible. **B**: The spanning tree includes only the minimal set of 38 links that connect all the nodes, but simultaneously maximize the sum of the link weights. **C**: The "double spanning graph" was formed by adding the links of a new spanning tree (constructed from the remaining links) on top the the first tree (38 + 38 = 76 links).

Measure of association for a heterogeneous pair of variables

For continuous data, one can simply use the Spearman correlation coefficient to quantify the link weights. However, binary data present a challenge since it has a much lower "information density" and linear dependence is an inefficient way to describe the statistical relationship between two binary traits or between continuous and binary variables. Therefore, an expanded measure that gave equal efficiency to every variable was developed.

For binary data, a Bayesian probit model was created for each variable, separately (V Johnson & J Albert (1999) Ordinal data modeling, Springer-Verlag New Work). Standard logistic regression was numerically unstable and could not be used. The target variable was estimated based on all the continuous variables in the dataset, and then the linear predictor of the model was stored as a surrogate continuous trait. Mathematically, the probit regression model estimates the probability of observing an event given

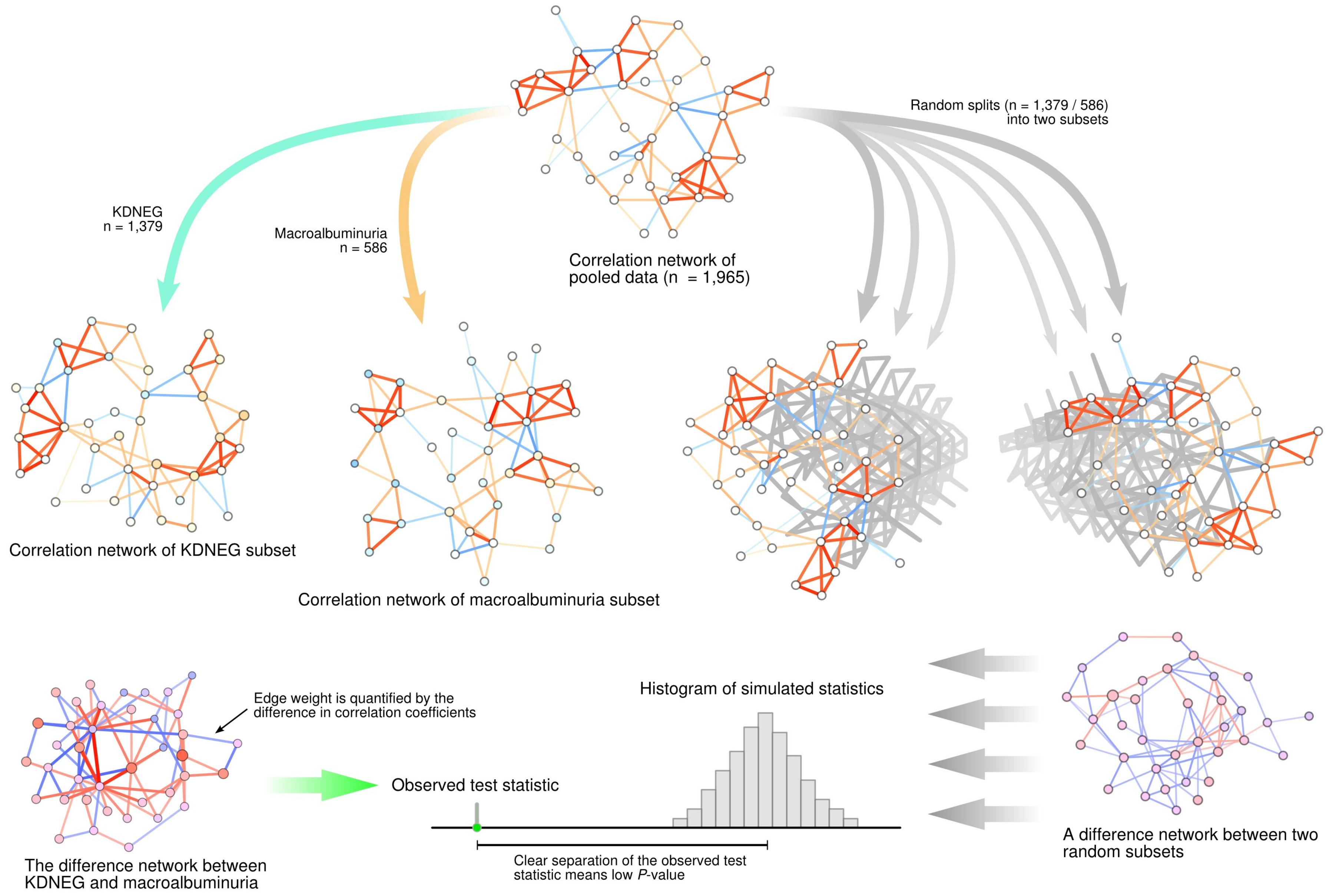

Figure S2: Schematic representation of the subset permutation analysis. The results are listed in Tables 1 and 2.

a linear combination of explanatory variables. Suppose the binary random variable Y represents a clinical event or diagnosis. The event probability can be estimated by $P(Y = 1 \mid \boldsymbol{x}) = f(\boldsymbol{xb})$ where $\boldsymbol{x}$ is the vector of continuous data observed for the patient, $\boldsymbol{b}$ is the vector of regression coefficients and $f()$ is the probit link function. The vector $\boldsymbol{b}$ is unknown *a priori* and is inferred from the dataset. The linear predictor $\boldsymbol{xb}$ is then used as a surrogate continuous variable for the original binary observation y.

The procedure was repeated for every variable (least-squares ridge-regression for the continuous) to ensure that the binary-binary links were comparable to binary-continuous and continuous-continuous links. This means that all the variables were replaced by linear combinations over the continuous variables. To reduce artificial inflation of correlation, the dataset was divided into two halves, one of which was then used to predict the other. After the binary and continuous data were converted to linear predictors, the network was constructed by calculating the correlation coefficients between the predictors instead of the observed values, which is referred to as "regression-correlation".

Visualization

Drawing a network on a two-dimensional canvas is an optimization problem, where one wishes to find such positions for the symbols (e.g. circles with the name of a variable attached) that the connecting lines cross as little as possible and that the figure is constrained within a reasonable area. For correlation networks with quantitative links, the coefficient magnitude should be reflected in the distance between two variables. Furthermore, not all links should be drawn, only the strongest and most essential should be visible to avoid clutter.

Here, the Himmeli software (http://www.finndiane.fi) was used to determine the symbol positions and to hide links selectively. The layout algorithm is a variant of the force-directed model, where the links are regarded as a set of connected springs that, when assembled together, will stabilize to an optimal configuration. The visible links are chosen by first calculating a spanning tree that maximizes the link strengths, then creating another spanning tree from the remainder, and so on until the desired level of connectedness is achieved (Figure S1). In this study, visually pleasing results were obtained by restricting the number of links to twice the number of variables.
