# Supplementary material for: Network of vascular diseases, death and biochemical characteristics in a set of 4,197 patients with type 1 diabetes (The FinnDiane Study)
Source: Cardiovasc Diabetol. 2009 Oct 6;8:54. doi: 10.1186/1475-2840-8-54 (PMC2763862; doi:10.1186/1475-2840-8-54)
Supplement: Additional file 3 — The Finnish Diabetic Nephropathy Study Group. A listing of the hospitals and health care centers that have participated in the recruitment of patients. [file 1475-2840-8-54-S3.PDF]

Additional file 3

The Finnish Diabetic Nephropathy Study Centers

Anjalankoski health care center	Sirpa Koivula
	Tarja Uggeldahl
Espoo health care center, Espoonlahti	Anita Nikkola
	Eila Ritola
Espoo health care center, Samaria	Eija Oukko-Ruponen
Espoo health care center, Tapiola	Hanna Saarinen
	Merja Kataja-Tuomola
	Marjatta Niska
Espoo health care center, Viherlaakso	Anne Lyytinen
	Satu Raumavirta-Koivisto
	Marjo Kovanen
Etelä-Karjala Central Hospital	Tuula Ensala
	Esko Hussi
	Jukka Toivonen
	Tuula Matikainen
	Raimo Vanamo
	Helga Hasu
Heinola health care center	Päivi Hentunen
	Jukka Lagerstam
Helsinki health care center, Puistola	Kari Pohjola
	Heli Silvennoinen
	Tiina Simonen
	Hannele Kari
Helsinki health care center, Suutarila	Anja Kaprio
	Birgitta Rantaeskola
Helsinki health care center, Töölö	Jari Haaga
	Pirjo Kääriäinen
	Anna-Liisa Pietiläinen

Helsinki Herttoniemi Hospital	Markku Saraheimo
	Kerstin Wickström
Helsinki University Central Hospital	Paula Nikkanen
	Pirkko Salmi
Hyvinkää health care center	Sisko Klemetti
	Teija Nyandoto
	Eija Rontu
	Susanna Satuli-Autere
Iisalmi Hospital	Eija Toivanen
	Jaana Hyytiäinen
Jokilaakso Hospital	Tuula Laitinen
	Jukka Lagerstam
Jorvi Hospital	Suvimarja Aranko
	Tarmo Leppälä
	Suvi Ervasti
	Ritva Kauppinen-Mäkelin
	Arto Kuusisto
	Katriina Nikkilä
	Leena Pekkonen
Jyväskylä health care center	Kirsti Nuorva
	Marja Tiihonen
Järvenpää health care center	Tuula Kinnunen
	Liisa Nikulainen
	Tuula Salokangas
Kainuu Central Hospital	Silja Jokelainen
	Paula Kemppainen
	Anna-Maria Mankinen
	Marja Sankari

	Tuula Myllykangas	Kuopio University Central Hospital	Erja Huttunen
Kanta-Häme Central Hospital	Pirjo Kinnunen		Risto Ikäheimo
	Anja Orvola		Päivi Kiljander
	Tapani Salonen		Markku Laakso
	Annukka Vähänen		Timo Lakka
Kerava health care center	Helene Stuckey		Leena Moilanen
	Paula Suominen		Leo Niskanen
Keski-Pohjanmaa Central Hospital	Stefan Anderson		Ulla Tuovinen
	Beatrice Asplund		Ilkka Vauhkonen
	Ulrika Byskata		Erkki Voutilainen
	Ingvor Byskata		Pauli Karhapää
	Toini Virkkala		Päivi Kekäläinen
	Päivi Liedes		Erkki Lampainen
Keski-Suomi Central Hospital	Terje Forslund	Kuusamo health care center	Erja Isopoussu
	Pirkko Koskiaho		Tapio Kääriäinen
	Marianne Laukkanen		Eero Vierimaa
	Juha Saltevo	Kuusankoski Hospital	Esa Kilkki
	Marja Tiihonen		Liisa Riihelä
	Pirkko Koskiaho	Kymenlaakso Central Hospital	Mare Riihelä
	Anne Halonen		Leena Ryysy
	Arvo Koistinen		Reino Paldanius
	Esa Leppänen	Laakso Hospital, Helsinki	Taina Meriläinen
	Anna-Mari Koski		Paula Poukka
Kirkkonummi health care center	Anja Lappalainen		Tuula Tikkanen
	Maisa Liimatainen		Riitta Savolainen
	Jaana Santaholma	Lahti City Hospital	Aulikki Mäkelä
Kivelä Hospital, Helsinki	Ari Aimolahti		Marjukka Tanner
	Maarit Kärki		Eija Behm
Koskela Hospital, Helsinki	Maija Lehtimäki	Länsi-Pohja Central Hospital	Pirkko Nyländen
	Nina Uhlenius		Hilkka Laukkanen
	Eeva Huovinen		Pirjo Myllykoski
	Veikko Ilkka	Länsi-Uusimaa Hospital	Inge-Maj Jousmaa
Kotka health care center	Eeva Pälikkö-Kontinen		Jukka Rinne
	Annika Vanhanen	Lappeenranta health care center,	Pirjo Linkola
Kouvola health care center	Erkki Koskinen	Armila Hospital	Irja Pulli
	Anna Borgman	Lappi Central Hospital	Sirkka Severinkangas
	Taimi Siitonen		Timo Tulokas

	Liisa Hyvärinen		Tarja Tick-Sinkkilä
Lohja Hospital	Tuula Granlund	Palokka health care center, Palokka	Leena Welling
	Mikko Saari	Palokka health care center, Vaajakoski	Kirsti Mäkinen
	Tuula Salonen		Päivi Sopanen
	Reija Tähtinen	Pieksämäki Hospital	Mari Tamminen
Loimaa health care center	Pirjo Eloranta		Markku Taskinen
	Arja Mäkelä	Pietarsaari Hospital	Maj-Len Holmbäck
Lounais-Häme Hospital	Tarja Kalliomäki		Bo Isomaa
	Juha Koskelainen		Leena Sarelin
	Riitta Nikkanen	Pohjois-Karjala Central Hospital	Laura Hyttinen
	Vesa Salonen		Päivi Kekäläinen
	Niilo Savolainen		Maija Pietarinen
	Hannu Sulonen		Anneli Rissanen
	Eija Valtonen		Risto Laitinen
Malmi Hospital, Helsinki	Helena Lanki		Hannu Turtola
	Seija Moilanen		Ulla Maija Henttula
	Marju Tilly-Kiesi	Pori City Hospital	Pirjo Merensalo
Mänttä Hospital	Anne-Mirjami Hänninen		Kirsi Sävelä
	Ismo Pirttiniemi		Terttu Viitala
Mikkeli Central Hospital	Anita Gynther		Päivi Ahonen
	Maarit Salminen	Porvoo Hospital	Heidi Johansson
	Tuomo Vänttinen		Sirkka Rämö
Nurmijärvi health care center	Mikko Honkasalo		Bjarne Rask
	Marko Miettinen		Marjatta Kallion
	Päivi Nurminen	Raahe Hospital	Anne Holma
Oulaskangas Hospital	Elina Jokelainen		Matti Honkala
	Pirkko-Liisa Jylkkä		Aino Tuomivaara
	Elsi Kaarlela		Riitta Vainionpää
	Jari Vuolaspuro	Rauma Hospital	Kaisa Laine
Oulu health care center	Rauni Häkkinen		Taru Salminen
	Liisa Hiltunen		Paula Ketola
	Sirkka Keinänen-Kiukaanniemi		Kaisa Saarinen
Päijät-Häme Central Hospital	Hannu Haapamäki	Riihimäki Hospital	Pirkko Aalto
	Satu Hämäläinen		Marja Lanni
	Antero Helanterä		Irmeli Laitaharju
	Vesa Ilvesmäki		Eija Immonen
	Hilkka Miettinen		Leena Juurinen

Salo Hospital	Jouni Lapinleimu		Heikki Oksala
	Pirkko Rautio		Tuija Oksanen
	Merja Virtanen		Kristiina Salonen
	Anita Alanko		Sirkku Tulokas
Satakunta Central Hospital	Markku Asola		Hilkka Tauriainen
	Paavo Pääkkönen	Tiirismaa health care center	Leena Petlin
	Marjo Pentti		Liisa Savolainen
	Marja Rautavirta		Tiina Kivelä
	Pertti Kunelius	Turku health care center	Anne Artukka
	Sirkku Mannila		Irmeli Hämäläinen
Savonlinna Central Hospital	Tuula Pulli		Päivi Kokkila
	Päivi Sallinen		Lea Sorvari
	Eija Toivanen		Markku Vähätalo
	Helena Valtonen		Hille Virtamo
	Aarne Vartia	Turku University Central Hospital	Kaija Breitholz
Seinäjoki Central Hospital	Eeva Korpi-Hyövälti		Raija Eskola
	Terhikki Latvala		Kaj Metsärinne
	Esko Leijala		Riikka Neva
	Tarja Nyrhilä		Ulla Pietilä
Tammisaari Hospital	Jukka Rinne		Pirjo Saarinen
	Inge-Maj Jousma		Riitta Tuominen
Tampere health care center	Pekka Alarotu		Markku Asola
	Liisa Calonius		Sari Äyräpää
	Seppo Gummerus	Vaajakoski health care center	Päivi Sopanen
	Paula Helin		Kirsti Mäkinen
	Tarja Kaitala	Vaasa Central Hospital	Stig Bergkulla
	Helena Kirkkopelto-Jokinen		Ulla Hautamäki
	Eila Kujansuu		Vivi-Ann Myllyniemi
	Taru Niskanen		Irma Rusk
	Atte Vadén	Valkeakoski Hospital	Terhi Immonen
	Timo Saaristo		Seppo Ojanen
	Marjo Kuortti		Maisa Rautiainen
	Arja Kallio		Eija Valtonen
Tampere University Central Hospital	Ilpo Ala-Houhala		Hannu Ylönen
	Tuula Kuningas	Vammala Hospital	Irma Isomäki
	Paula Lampinen		Leena Mustaniemi
	Marita Määttä		Merja Tapiolinna-Mäkela

	Riitta Kroneld
Vantaa health care center, Korso	Jaana Vahtola
	Riitta Toivonen
Vantaa health care center, Länsimäki	Raila Ahonen
	Marja Ivaska-Suomela
	Anu Jauhiainen
	Sari Kultti
	Pirjo Kiviniemi
Vantaa health care center, Martinlaakso	Merja Laine
	Tiina Pellonpää
	Riitta Puranen
Vantaa health care center, Myyrmäki	Anne Airas
	Jukka Laakso
Vantaa health care center, Rekola	Matti Eerola
	Eeva Jatkola
Vantaa health care center, Tikkurila	Riitta Lönnblad
	Jari Mäkelä
	Anita Malm
	Elli Rautamo
Åland Central Hospital	Hans Granlund
	Ann-Christin Johnsson
	Monica Forsen
Äänekoski health care center	Satu Savolainen
	Tapani Marjanen
